# Humic acid enhances heat stress tolerance via transcriptional activation of Heat-Shock Proteins in Arabidopsis

**DOI:** 10.1038/s41598-020-71701-8

**Published:** 2020-09-14

**Authors:** Joon-Yung Cha, Sang-Ho Kang, Imdad Ali, Sang Cheol Lee, Myung Geun Ji, Song Yi Jeong, Gyeong-Im Shin, Min Gab Kim, Jong-Rok Jeon, Woe-Yeon Kim

**Affiliations:** 1 Division of Applied Life Science (BK21Plus), Plant Molecular Biology and Biotechnology Research Center (PMBBRC), Research Institute of Life Sciences (RILS), Gyeongsang National University, Jinju, 52828 Republic of Korea; 2 Genomics Division, National Institute of Agricultural Sciences, Rural Development Administration, Jeonju, 54874 Republic of Korea; 3 College of Pharmacy and Research Institute of Pharmaceutical Science, PMBBRC, Gyeongsang National University, Jinju, 52828 Republic of Korea; 4 Department of Agricultural Chemistry and Food Science & Technology, Institute of Agriculture and Life Science (IALS), Gyeongsang National University, Jinju, 52828 Republic of Korea

**Keywords:** Computational biology and bioinformatics, Molecular biology, Plant sciences

## Abstract

Humic acid (HA) is composed of a complex supramolecular association and is produced by humification of organic matters in soil environments. HA not only improves soil fertility, but also stimulates plant growth. Although numerous bioactivities of HA have been reported, the molecular evidences have not yet been elucidated. Here, we performed transcriptomic analysis to identify the HA-prompted molecular mechanisms in Arabidopsis. Gene ontology enrichment analysis revealed that HA up-regulates diverse genes involved in the response to stress, especially to heat. Heat stress causes dramatic induction in unique gene families such as *Heat-Shock Protein* (*HSP*) coding genes including *HSP101*, *HSP81.1*, *HSP26.5*, *HSP23.6*, and *HSP17.6A*. HSPs mainly function as molecular chaperones to protect against thermal denaturation of substrates and facilitate refolding of denatured substrates. Interestingly, wild-type plants grown in HA were heat-tolerant compared to those grown in the absence of HA, whereas Arabidopsis *HSP101* null mutant (*hot1*) was insensitive to HA. We also validated that HA accelerates the transcriptional expression of HSPs. Overall, these results suggest that *HSP101* is a molecular target of HA promoting heat-stress tolerance in Arabidopsis. Our transcriptome information contributes to understanding the acquired genetic and agronomic traits by HA conferring tolerance to environmental stresses in plants.

## Introduction

Humic acid (HA), a main component of humic substances (HSs), is composed of various kinds of organic components forming a complex supramolecular association and the material is widespread in the environment, wherein organic matter mainly involving plants is humified^1,2^. Detailed structures of HSs depend on their extraction sources such as soils, rivers and coal-related materials, but it is generally accepted that HSs contain both aromatic and aliphatic components displaying several organic functional groups^3,4^. Based on their structural features, HSs has prompted their agricultural use as fertilizer, and it has been reported that HSs not only improve soil fertility by increasing water-holding and cation exchange capacities, but also stimulate plant growth through enhanced nutrient uptake and phytohormone-like activities^4–6^. It was experimentally confirmed that humic components are able to penetrate into plant tissues, thus triggering activation of versatile signaling and metabolic pathways and changing physiological processes for plant growth and development^7,8^.

Recent climate changes due to global warming cause various abiotic stresses including abnormal temperature, salt, and drought stresses that are further connected to adverse impacts in crop productivity. Heat, a major abiotic stress of plants by global warming, incurs irreversible damages hampering plant photosynthesis, development and reproduction^9–11^. When exposed to a heat stress, ubiquitous HEAT-SHOCK PROTEINs (HSPs) genetically expressed by all living organisms are prone to be upregulated on the transcriptional level and act as a molecular chaperone to protect thermal degradation of proteins and to facilitate the renaturation of non-native proteins, helping in the maintenance of protein homeostasis under stress conditions^12,13^.

HSPs are classified as their approximate molecular masses such as HSP100, HSP90, HSP70, HSP60, and small HSP (ranging between 15–42 kDa), and distributed in various subcellular compartments^12^. HSPs are mainly involved in heat stress tolerance, and help to maintain large client proteins in soluble state functioning properly in cell^13^. Protein denaturation is common consequence of diverse stresses including extreme temperatures, drought, salt, UV, and pathogen infection, and the process could be hindered by chaperone modules of various HSPs, resulting in mitigating negative impacts of environmental stresses^13^. Beyond the stress defense roles, it has been also identified that the expression levels of HSPs critically affect several plant physiological phenomena such as seed germinations, growth and pollen development in the absence of stress^14–16^, supporting the notion that HSPs act as a global regulator controlling several biological pathways.

We recently provided a key evidence on how HA can activate plants to tolerate a salt-related abiotic stress through the post-transcriptional regulation of HIGH-AFFINITY K + TRANSPORTER 1^17^. This result strongly indicates that specific molecular targets controlled by HA are present. Although multifunctional biological effects of HA and HSs in several species of plants have been proved^6,8^, the global genetic changes at the molecular level in response to HA have not yet been elucidated. Here, we thus performed a transcriptomic analysis to elucidate the molecular targets of HA and to identify their molecular mechanisms in which plants tolerate a heat-related stress. Gene Ontology enrichment analysis revealed that HA up-regulates genes encoding various HSPs, and thus HA has a priming protective effect against heat stress in Arabidopsis. A genetic mutant of Arabidopsis was also employed to confirm whether the target genes are sensitive to the HA treatments. Understanding the overall alteration of gene transcription will provide genetic evidence to extend the agronomical importance of HA.

## Results

### Illumina sequencing and mapping sequence reads, and total differentially expressed genes (DEG)

HSs are able to upgrade soil fertility based on their physicochemical interactions with water, metallic plant nutrients and soil particles, and besides, it was experimentally confirmed that some organic components in HSs have a potential to infiltrate plant tissues followed by direct modulation of signaling and metabolic pathways at both the transcriptional and post-transcriptional levels^7,8,18^. It could be also reasoned that HSs act as a global gene regulator since several physiological actions of plants including nutrient uptake, lateral root development, stomatal response, and abiotic stress tolerance, have been proven to be inducible in the presence of HSs^8,17,19–22^.

To identify target genes that can be controlled by HA, we performed transcriptomic analyses in Arabidopsis whose wild-type seedlings were grown on MS medium for 7 days, and they were transferred onto a MS medium in the absence (MS) or presence of HA (860 mg L^−1^) for 9 h to see the dynamic changes. The transcriptional expression of *INDOLE-3-ACETIC ACID INDUCIBLE19* (*IAA19*) was used to validate whether the above HA treatment condition stimulates Arabidopsis because it was previously proven that HSs induce the acceleration of lateral root formation with the expression of the early auxin-responsive *IAA19* gene^23^. Consistent with the previous phenomena, the significant up-regulation of *IAA19* was here identified (Supplementary Fig. S1).

To provide a comprehensive overview of HA-induced biological activities in Arabidopsis at the transcriptional level, we sequenced cDNA libraries constructed for each treatment by using an Illumina HiSeq 2,500 platform. A total of 320,226,692 sequence reads were produced from both treatments (MS and HA) with three biological replications. On average, 94.3% of the quality-filtered reads generated from all the samples were uniquely mapped to the reference genome; the other reads were either unmapped (4.3%) or did not show primary hits (1.4%). A summary of mapping statistics obtained from each treatment was described in Supplementary Table S1. Transcriptional changes in Arabidopsis seedlings exposed to MS or HA were comparatively analyzed to identify the DEGs (Additional file 1).

### Genes transcriptionally up-regulated by HA application are enriched for the ‘response to stimulus’ gene ontology (GO) term

Of 3,257 DEGs (log_2_ fold change ≥ 0.3 and cutoff q-value < 0.05) between MS and HA treatments, 1,677 genes were significantly up-regulated by HA while 1,580 genes were significantly down-regulated. From these large numbers of DEGs, we re-analyzed DEGs showing log_2_ fold change ≥ 1 and ≤ − 1 for up- and down-regulated genes, respectively. Among these re-analyzed DEGs, 416 genes were up-regulated by HA while 347 genes were down-regulated (Additional file 2).

**Figure 1:**
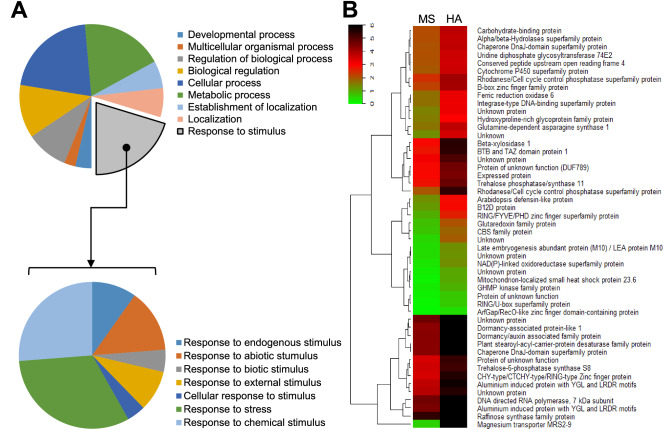
Gene Ontology (GO) classification based on biological process and the top50 DEGs. (**A**) Biological processes significantly up-regulated genes by HA compared to MS (*top*) and sub-classification of DEGs involved in the response to stimulus (*bottom*). (**B**) Heat maps of the top 50 DEGs in the up-regulated genes by HA. Gene expression levels were clustered based on the log2 (FPKM + 1).

The 416 DEGs up-regulated by HA were investigated for GO term enrichment^24^, with 21% of the 416 DEGs significantly enriched (False Discovery Rate, FDR = 4.2e−05) in the ‘response to stimulus’ GO category related to biological process (GO:0008150); however, genes involved in the molecular function (GO:0003674) and cellular component (GO:0005575) categories were not significantly enriched (FDR = 1) (Fig. 1A and Supplementary Fig. S2). In the GO sub-categories under the GO term ‘response to stimulus’ (GO:0050896), 11 genes were associated with the ‘response to external stimulus’ category (GO:0009605; FDR = 0.012), 39 genes were associated with ‘response to stress’ (GO:0006950; FDR = 4.2e−05), and 32 genes were associated with ‘response to chemical stimulus’ (GO:0042221; FDR = 0.0022) (Fig. 1A). The 50 most up-regulated genes are shown in Fig. 1B. These genes mainly involve in the response to stress and chemical stimulus, which are identical with entire DEGs up-regulated by HA (Fig. 1). These data strongly suggest that even when plants are not exposed to stress conditions, HA application itself activates transcription of stress-related genes that contribute to environmental stress tolerance.

**Figure 2:**
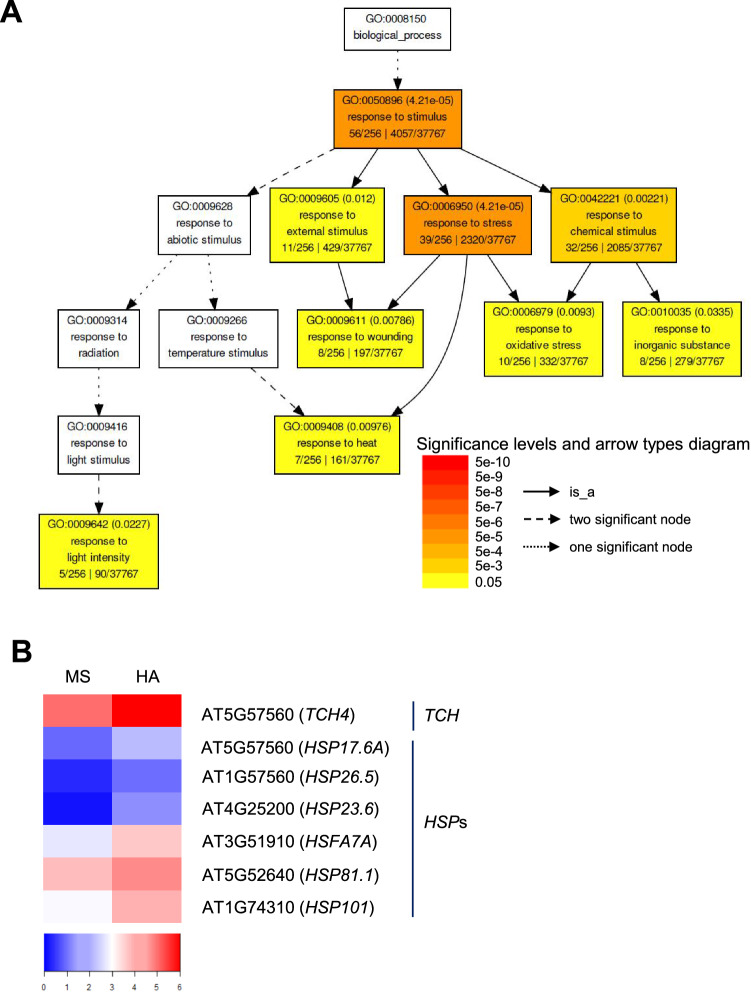
GO term enrichment analysis of biological process and the DEGs in response to heat term. (**A**) GO term enrichment analysis for up-regulated genes by HA. Each box indicates the GO term and description with the FDR-adjusted *p*-value; the color scale reflects these adjusted *p*-values. The fraction on the left side at the bottom is the number of genes in our dataset falling into that GO category out of the total number of genes in the list. Boxes with GO terms are presented hierarchically, with the root term at the *top* and child terms toward the *bottom*. (**B**) Heat maps of the DEGs involved in ‘response to heat’ GO category. Expression values were log2-transformed and then median-centered by variant.

The lists of 56 DEGs involved in response to stimulus GO category were investigated for GO term enrichment of biological processes (Fig. 2A). Genes involved in responses to wounding (GO:0009611), oxidative (GO:0006979), and heat stress (GO:0009608) were significantly enriched (FDR < 0.01) (Fig. 2A). The genes in ‘response to wounding’ category were *WOUND-INDUCED POLYPEPTIDE 3* (*WIP3,* AT4G10265), *WIP4* (AT4G10270), *SENESCENCE 1* (*SEN1*, AT4G35770), *12-OXOPHYTODIENOATE REDUCTASE 1* (*OPR1*, AT1G76680), *CALMODULIN-LIKE 38* (*CML38*, AT1G76650), and *BIFUNCTIONAL NUCLEASE IN BASAL DEFENSE RESPONSIVE 2* (*BBD2*, AT1G19660), and they share responses with pathogen attack including viral and bacterial infections^25–29^. Genes including *PEROXIDASE* (*PRX/PRX65*, AT5G47000), *26.5 kDa CLASS 1 SMALL HSP-LIKE* (*HSP26.5-P*, AT1G52560), *SEN1*, *DEHYDRATION-RESPONSIVE ELEMENT-BINDING PROTEIN 2A* (*DREB2A*, AT5G05410), *ARABIDOPSIS ORTHOLOG OF SUGAR BEET HS1 PRO-1 2* (*HSPRO2*, AT2G40000), and *OXIDATIVE STRESS 3* (*OXS3*, AT5G56550) involved in the ‘response to oxidative stress and hydrogen peroxide’ categories were also up-regulated by HA. Peroxidase is an antioxidant enzyme that scavenges toxic H_2_O_2_ producing H_2_O. Interestingly, *PRX65* is expressed only in roots and not in other plant parts including stems, flowers, and leaves^30^. This raises the possibility that HA positively regulates responses to abiotic stress originating from the soil, such as salinity and heavy metal stress^31^. Overexpression of *DREB2A* or *OXS3* in Arabidopsis confers tolerance to diverse abiotic stresses such as drought, cold, heavy metals and oxidative stresses^32,33^. In the ‘response to heat’ category, *HEAT-SHOCK PROTEIN* (*HSP*) *101* (*HSP101*, AT1G74310), *HSP81.1* (AT5G52640), *HSP26.5-P*, *SMALL HSP 17.6A* (*HSP17.6A*, AT5G12030), *MITOCHONDRION-LOCALIZED SMALL HSP 23.6* (*HSP23.6-MITO*, AT4G25200), and *HS TRANSCRIPTION FACTOR A7A* (*HSFA7A*, AT3G51910) known to be induced by heat stress were also up-regulated by HA (Fig. 2B). HSPs function as molecular chaperones to protect against thermal denaturation of substrates and facilitate refolding of denatured substrates^13,34^. In addition, large number of genes encoding HSPs and HSFs are induced not only by heat, but also by diverse biotic and abiotic stresses including cold, osmotic, salt, drought, wounding, pathogen, and oxidative stresses^35^. *TOUCH 4*/*XYLOGLUCAN ENDOTRANSGLUCOSYLASE AND HYDROLASE 22* (*TCH4*/*XTH22*, AT5G57560), which is activated by diverse stimuli including touch, cold, and heat, is also induced by HA application^36^. These genes up-regulated by HA in the ‘response to stress’ GO category suggest that HA activates diverse gene sets involved in stress defense mechanisms.

### Validation of up-regulated genes by qRT-PCR (HA transcriptionally enhances HSP genes)

**Figure 3:**
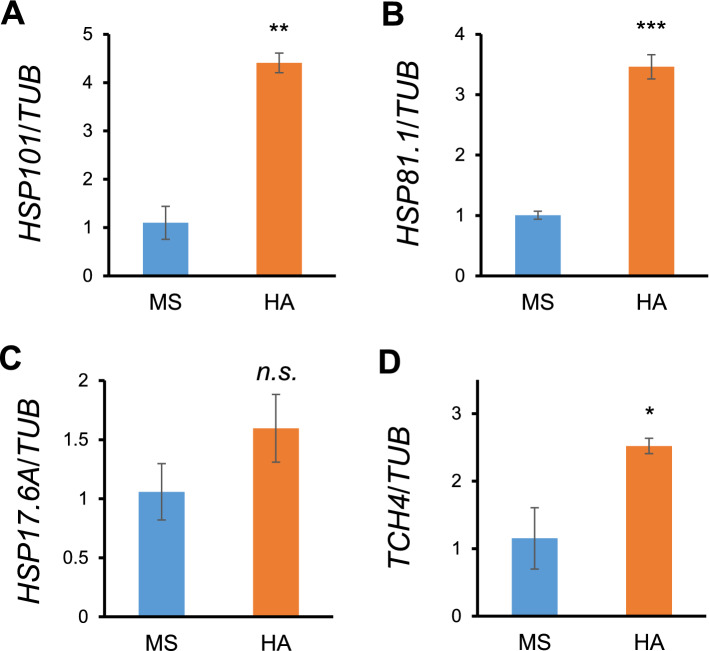
Expression validations of heat-inducible DEGs up-regulated by HA. One-week old Arabidopsis wild-type (Col-0) plants were treated in the absence or presence of humic acid (860 mg L^−1^) for 9 h, and validated by qRT-PCR. Transcripts of *HSP101* (**A**), *HSP81.1* (**B**), *HSP17.6A* (**C**), and *TCH4* (**D**) were normalized to tubulin. Data represent means ± SE of three independent biological replicate experiments. Significant differences are shown as asterisks (Student *t*-test, *P < 0.05; **P < 0.01; ***P < 0.001; *n.s.*, no significant).

To validate results of the transcriptomic analysis, expression of four genes that were up-regulated by HA was examined by qRT-PCR. First, four genes including three *HSP* genes (*HSP101*, *HSP81.1*, and *HSP17.6A*) and *TCH4* were analyzed, and results showed that all genes examined were up-regulated by HA, which is consistent with transcriptomic analysis (Fig. 3). HSP and HSF genes are not only induced by heat stress, but also by other types of environmental stresses such as cold, salt, and osmotic stress^35^. Thus, HA activates transcription of HSP genes even in the absence of stressful factors, suggesting that HA applications to plants may confer heat stress tolerance.

### HA confers thermotolerance via HSP101 as a molecular target of HA in Arabidopsis

**Figure 4:**
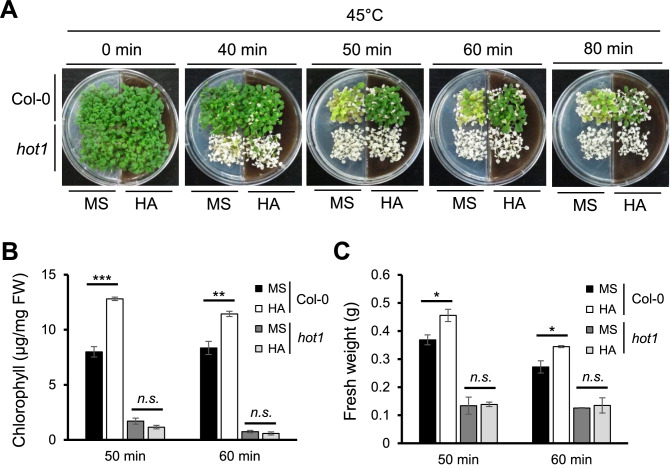
Humic acid enhances thermotolerance in Arabidopsis via *HSP101* as a molecular target of HA. Two-week old Arabidopsis wild-type (Col-0) and HSP101 mutant (*hot1*) grown in the absence or presence of humic acid (860 mg L^−1^) were treated at 37 °C for 1 h for heat-acclimation, transferred to room temperature (23 °C) for 2 h, and finally subjected to 45 °C for indicated time. Pictures (**A**) were taken one week after heat treatment, and plants were immediately used for measuring chlorophyll contents (**B**) and fresh weight (**C**). Data are means ± SE of triplicate biological replications. Significant differences are shown as asterisks (Student *t*-test, *P < 0.05; **P < 0.01; ***P < 0.001; *n.s.*, no significant).

To assess whether HA drives physiological responses under heat stress based on transcriptomic analysis, we used two-week-old Arabidopsis plants grown in the absence (MS) or presence of HA. For monitoring the acquired heat stress tolerance, Arabidopsis plants were pre-incubated at 37 °C for 1 h as heat-acclimation, rested at 23 °C for 2 h, and exposed to severe heat stress at 45 °C for indicated periods. Wild-type plants grown in the presence of HA showed dramatic tolerance with healthy green leaves to heat stress at 45 °C, while those grown in the absence of HA became pale green and retarded growth as processing time increase under heat stress (Fig. 4A). Heat stress inhibits photosynthetic activity by damaging in metabolic processes such as chlorophyll breakdown, and consequently reduces biomass^11^. Thus, we measured chlorophyll contents and fresh weight as physiological parameters using the plants exposed to heat stress at 45 °C for 50 and 60 min. Both chlorophyll content and fresh weight of wild-type plants after heat stress were significantly higher in plants grown HA compared to those grown in MS (Fig. 4B,C). It suggests that HA confers thermotolerance in Arabidopsis, and its application may help plants to escape from extreme heat stress.

HSPs are highly conserved in all living kingdoms, and each HSP family is encoded by multi-gene family^35^. In Arabidopsis, there are eight isoforms of HSP100, seven HSP90, eighteen HSP70, and thirteen HSP20. In addition, twenty-one HSFs were identified to regulate the transcription of HSP genes^35^. Due to these large gene family of HSPs, there are functional redundancy between isoforms causing disable to identify individual gene using mutant analysis^37,38^. Interestingly, mutations in Arabidopsis *HSP101* (*hot1*) abolished thermotolerance, but showed no morphological differences compared to wild-type in the absence of heat stress^39^. Thus, we also examined whether the heat-sensitivity of *hot1* mutant is rescued by HA. As same as heat stress assay using wild-type plants, *hot1* mutants grown in the absence or presence of HA were exposed to heat stress. Interestingly, we found that *hot1* mutants were extremely sensitive to heat stress compared to wild-type and unable to survive both in the absence and presence of HA under heat stress conditions (Fig. 4A). Both physiological parameters of heat stress responses such as chlorophyll contents and fresh weight in *hot1* mutants were dramatically decreased by heat stress, however they did not show any significant differences between MS and HA (Fig. 4B,C). Thus, *hot1* mutants are insensitivity to HA conferring thermotolerance in Arabidopsis, suggesting that *HSP101* is a molecular target of HA. We also performed basal thermotolerance assay. Two-week old wild-type and *hot1* plants were directly exposed to heat stress at 45 °C without heat-acclimation. However, survivability and chlorophyll contents were not significantly different between the plants grown in MS and HA media (Supplementary Fig. S3). Collectively, HA enhances heat tolerance of heat-acclimated plants.

### HA promotes early activation of HSPs under heat stress

**Figure 5:**
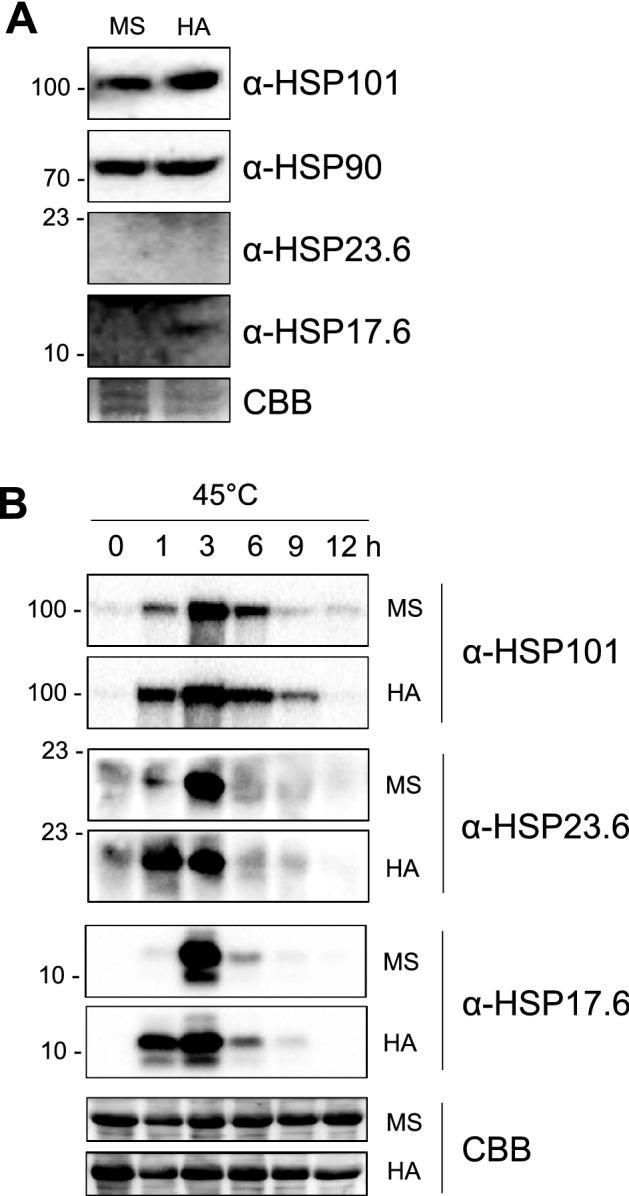
HA triggers expression of HSP proteins. Two-week old Arabidopsis wild-type (Col-0) plants grown in the absence or presence of humic acid (860 mg L^−1^) were harvested in the absence (**A**) or presence of heat stress (45 °C, **B**), and carried immuno-blot analysis using α-HSP101, α-HSP23.6, and α-HSP17.6. Coomassie brilliant blue (CBB) stained blot was used as a loading control. Triplicate biological replications were performed with consistent results.

Next, we examined protein levels of HSPs. Two-week old Arabidopsis wild-type plants grown in the absence or presence of HA were analyzed by immuno-blot analysis using anti-HSPs antibody. Interestingly, protein levels of HSP101 and HSP17.6 were slightly induced by HA applications, while HSP23.6 did not detect both in the absence or presence of HA (Fig. 5A). In addition, HSP90 did not show difference between MS and HA in the absence of heat stress (Fig. 5A). Transcriptional and translational levels of HSPs as stress proteins are dramatically enhanced under stress conditions, especially heat stress, and consequently, they determine heat stress tolerance in plants^40^. Thus, we examined whether HA enhances expressions of HSPs under heat stress condition. Two-week old Arabidopsis wild-type plants grown MS or HA media were exposed to heat stress at 45 °C and analyzed by immuno-blot analysis. Interestingly, expression levels of HSP101, HSP23.6, and HSP17.6 were rapidly increased in the presence of HA compared to those in the absence of HA (Fig. 5B). All HSPs examined in this study were peaked at 3 h after heat treatment in MS, however they were highly induced from 1 h after the treatment and peaked at 3 h in HA. In addition, expression of HSP101 was significantly higher in the presence of HA under heat stress compared to that in the absence of HA (Fig. 5B; Supplementary Fig. S4). However, HSP90 did not show the difference between MS and HA treatments (Supplementary Fig. S5). Thus, up-regulation of HSP transcripts by HA caused protein expression levels of HSPs, suggesting that HA transcriptionally enhanced HSPs, which are essential for heat stress tolerance in Arabidopsis.

## Discussion

Beyond the broad range of bioactivities, it has been suggested that HSs trigger diverse molecular processes in plants^8^. As major soil-borne organic matters, HSs largely affect to soil environments via increasing fertility and nutrient bioavailability, and positively regulate growth of root parts^6,41^. Interestingly, many studies including our previous reports have also found that foliar applications of humic matters increased plant yields in both shoot and root parts, suggesting that HSs trigger molecular processes at cellular levels causing physiological responses^8,42,43^. Recently, radio-labelled HA assay provided an evidence which HA moves into plant tissues^7^. When tritium-labeled leonardite HA fed to wheat and cucumber seedling, labelled products found mostly in the roots with smaller portion in the shoots, and also detected in the stele and xylem tissues^7^. Thus, HSs and HA can directly stimulate transcriptional, translational, and post-translational processes in plant tissues and manifest their biological activities in plant^8^.

A few evidences about potential molecular targets of HA have been reported in lateral root development, which is largely responsible for auxin. In maize, *Mha2*, a major isoform of H^+^-ATPase transcriptionally activated by auxin, is significantly induced by earthworm low molecular size HS^20,44^. It also has been reported that HA plays as similar as auxin in maize root development and activation of plasmalemma and tonoplast H^+^ pumps^45^. However, Schmidt et al.^46^ found that water-extractable fraction of HSs derived from sphagnum peat mainly affected to an ordered remodeling of the root morphology via the down-regulation of negative regulator genes involved in the root hair cell fate without auxin-like manner. In addition, calcium signaling is elucidated as a target of HS via activation of Ca^2^^+^-dependent protein kinase promoting root development in rice^47^. Cucumber treated by leonardite HA had increased iron uptake and assimilation via induction of *CsFRO1* and *CsIRT1*, encoding a Fe(III) chelate-reductase and a Fe(II) root transporter, respectively^48^. Thus, it is evidenced that HA and HSs may trigger cell division and growth in mitotic sites of lateral root^6,45^. Recently, Shah et al.^8^ have extensively discussed about molecular regulatory processes of HSs in plants. They pointed that the processes driven by HSs activate diverse downstream responses to strengthen the plant fitness against various kinds of abiotic stresses, however precise molecular mechanisms by HA and HSs are largely unknown due to comprehensive data are still missing^8^. Most recently, Nunes et al.^18^ tried proteomic analysis to understand the molecular mechanisms of HA-induced maize root development, and they found that proteins involved in energy metabolism, protein folding, cytoskeleton organization, RNA processing, stress response, N assimilation, transport of protein and vesicle transport were changed by HA. Although the putative molecular target proteins of HA had been suggested, it still remains elusive without any genetic, molecular, and physiological evidences.

To elucidate molecular targets of HA in plants, we carried out the transcriptomic analysis in Arabidopsis. Total 3,257 genes were differently regulated by HA including 1,677 up-regulated genes and belonged to diverse biological processes (Fig. 1A), raising the possibility that HA may play vital roles triggering various biological and physiological outputs in plants. Indeed, up-regulated genes by HA were identified to be responded in various internal and external stimulus with highly enriched DEGs in the ‘response to stress’ (Figs. 1B, 2A). Thus, our transcriptomic data could provide molecular mechanisms of HA conferring tolerance to various kinds of biotic and abiotic stresses in plants^8,49^. In this study, we focused on ‘response to heat’, because distinct subset of HSP coding genes was enriched in the up-regulation by HA and validated by qRT-PCR (Figs. 2B, 3). HSPs play as molecular chaperones protecting cells against thermal damage, and are essential for thermotolerance in plants^13^. In addition, proteomic approach also revealed that putative HSP90 family protein was enhanced in the plant roots grown with HA^18^. HSP100 and HSP90 family proteins play as a major ATP-dependent molecular chaperone repairing denatured proteins for refolding, and are essential for maintaining protein homeostasis in the cells^50^. Small HSPs act as an ATP-independent molecular chaperone preventing misfolding and irreversible aggregation of client proteins, and have crucial roles for acquiring tolerance to various abiotic stresses as well as plant development^37,51^. To activate the transcription of HSPs, heat-shock transcription factors (HSFs) recognize the heat stress elements in promoters of HSP genes in response to various abiotic stresses, and help plants to acquire the stress tolerances^52^. These genetic and biochemical evidences of HSPs and HSF raised the possibility that HA could confer heat stress tolerance in Arabidopsis. Indeed, we confirmed that Arabidopsis wild-type plants grown in the presence of HA were significantly tolerant to heat stress compared to those grown in the absence of HA under acquired thermotolerance test (Fig. 4). Interestingly, the HA-induced thermotolerance is not reenacted in the *HSP101* mutant *hot1* (Fig. 4). In the basal thermotolerance test, HA did not confer the heat tolerance both in wild-type and *hot1* plants (Supplementary Fig. S3). Exposure of heat-acclimation or preconditioning at mild heat stress enables plants survive against lethal temperatures^53^. And HSP101 has been known as a major regulator in acquired thermotolerance^39,53,54^. Immuno-blot analysis revealed that HA accelerates induction of HSP proteins (Fig. 5). Collectively, it indicates that HA enhances heat stress tolerance via transcriptional activation of HSPs, and furthermore, HSP101 is a molecular target of HA in Arabidopsis. However, there are still possibility which other HSPs (including HSP90 and small HSPs) and HSFA7A could be targets of HA. Each family of HSPs and HSFs is encoded by multigene family which shares functional redundancy^37,38^. In addition, mutations in *HSP101* (*hot1*) had been only identified with heat-sensitive phenotype among our HA-responsible candidate genes^39^. Thus, if genetic mutants showing heat-sensitivity are available, we could further verify whether our candidate genes are real targets of HA. However, it is clear that HA triggers expressions of HSPs both in the absence and presence of heat stress, and their enhanced chaperone capacity may protect numerous clients from thermal aggregation.

**Figure 6:**
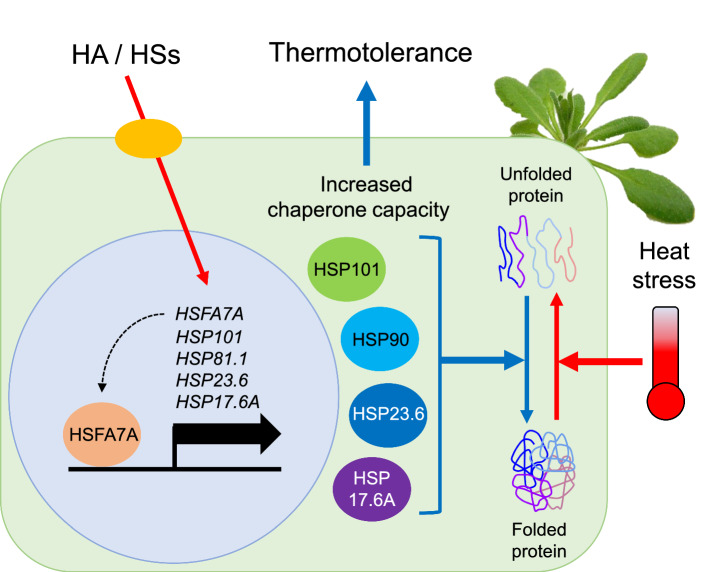
Proposed model of acquiring thermotolerance in plants by HA applications. HA applications to plants trigger the transcriptional expressions of HSP and HSF genes via unknown receptors. HA-induced HSFA7A may promote the transcriptional activation of HSP genes, and produced HSP proteins inhibit thermal denaturation of cellular proteins through their increased chaperone capacity. Consequently, HA-induced chaperone proteins confer heat stress tolerance in plants.

In conclusion, we conducted transcriptomic analysis to understand the unknown molecular mechanisms of HA in Arabidopsis. Transcripts of genes related to responses to stimuli were significantly enriched with diverse abiotic stress-related genes, especially HSP genes, up-regulated by HA. Interestingly, we found that HA confers thermotolerance in Arabidopsis via transcriptional activation of HSP genes (Fig. 6). We also concluded that HSP101 is a molecular target of HA. Thus, HA triggers an overall alteration of gene expression involved in abiotic stress tolerance, and thus confers heat stress tolerance in Arabidopsis. These novel findings provide genome-scale molecular evidence for bioactivity driven by HA, and will contribute to increasing the environmental adaptability of field crops to increase yield under worldwide global warming.

## Materials and methods

### Plant materials and growth conditions

For RNA-Seq analysis, seeds of *Arabidopsis thaliana* (Col-0 ecotype background) were surface-sterilized and grown on 1/2 MS medium for 7 days. Seedlings were then transferred to medium with or without 860 mg L^−1^ HA [Sigma Aldrich (Cat No#53680), MO, USA] for 9 h (from Zeitgeber time 1 to 10). Triplicate biological replications were prepared for RNA-Seq analysis. All plants were grown at 22 °C under 16 h light (100 μmol photons m^−2^ s^−1^)/8 h dark cycle. After harvesting, samples were immediately placed in liquid nitrogen and stored at − 80 °C until RNA extraction.

### RNA extraction and Illumina RNA-Seq

Total RNA was extracted from three biological replicates using an RNeasy Plant Mini Kit (Qiagen, MD, USA) according to the manufacturer’s instructions. Libraries for 100 bp paired-end sequencing were prepared using a TruSeq RNA Library Preparation kit (Illumina, CA, USA). mRNA purification, cDNA synthesis, and cDNA library construction have been reproduced in part from Park et al.^55^. Quality of the cDNA libraries was assessed with an Agilent 2,100 BioAnalyzer (Agilent, CA, USA), and quantification of them was carried using a KAPA library quantification kit (Kapa Biosystems, MA, USA). Subsequently, paired-end (2 × 100 bp) sequencing was performed using an Illumina HiSeq 2,500 sequencer (Illumina, CA, USA).

### Analysis of RNA-Seq data

Filtering of low-quality reads was performed as described previously^55^. For sequence alignment, filtered reads were mapped to the Arabidopsis (TAIR10) reference genome (https://www.arabidopsis.org/) using the aligner TopHat v2.0.12^56^. The Fragments Per Kilobase of exon per Million fragments mapped (FPKM) method was used to calculate the abundance of gene expression with Cufflinks v2.1.1^57,58^. Multi-read-correction and frag-bias-correct options were applied to enhance the measurement accuracy. The plant transcriptional factor database v3.0 (PlantTFDB, https://planttfdb.cbi.pku.edu.cn/) was used to identify various transcription factors among DEGs. All DEGs with TAIR10 identifiers were used as input for search against PlantTFDB.

### DEG and GO analysis

DEGs were identified based on a q-value threshold less than 0.05 for correcting errors caused by multiple testing^59^. GO enrichment analysis with the DEGs based on log_2_ fold change values ≥ 1 was carried out using agriGO v1.2^24^. GO-based trend test was carried out through the Fisher’s exact test^60^ to characterize the genes identified from DEG analysis.

#### Heat stress treatments

For acquired thermotolerance assay with heat-acclimation, two-week old Arabidopsis wild-type (Col-0) and *HSP101* null mutant (*hot1*) grown in the absence or presence of HA (860 mg L^−1^) were acclimated at 37 °C for 1 h, followed by 23 °C for 2 h, and exposed to heat stress at 45 °C for indicated time points. For basal thermotolerance assay without heat-acclimation, two-week old plants grown with or without HA were directly exposed to heat stress at 45 °C for indicated time points. Fresh weight and chlorophyll content were measured at 5 days after heat stress treatments with three independent biological replications^61^.

#### Validation by quantitative reverse-transcription PCR (qRT-PCR)

Seven-day old Arabidopsis (Col-0) seedlings grown on 1/2 MS medium were treated with or without HA (860 mg L^−1^) for 9 h, and were frozen in liquid nitrogen immediately. Total RNA extraction, cDNA synthesis, and qRT-PCR analysis were carried as described previously^62^. The PCR was performed as follows: 95 °C for 5 min; 40 cycles of 95 °C for 30 s, 58 °C for 45 s, and 72 °C for 45 s; followed by 72 °C for 5 min. The expression level of target genes was normalized to that of the housekeeping gene, *TUBULIN* (*TUB*), via the delta-delta Ct (ΔΔCt) method. Triplicate biological replications were performed. The primer sequences used in this study are listed in Supplementary Table S2.

#### Immunoblot analysis

Total proteins were extracted in the extraction buffer as described previously^63^, and separated on SDS-PAGE. Immunoblot analysis was performed using α-HSP101 (1:30,000, Agrisera, Sweden), α-HSP23.6 (1:2,000, Agrisera, Sweden), and α-HSP17.7 (1:20,000, Agrisera, Sweden). The antigen protein was detected by chemiluminescence using ECL-detecting reagent (Thermo Scientific, IL, USA). Triplicate biological replications were performed with consistent results.

## Supplementary information

Supplementary Information 1.

Supplementary Information 2.

Supplementary Information 3.

## Data Availability

The sequence data generated in this study have been deposited at the National Center for Biotechnology Information (NCBI) Sequence Read Archive database with the accession number SUB7353892.
