# Supplementary material for: Humic acid enhances heat stress tolerance via transcriptional activation of Heat-Shock Proteins in Arabidopsis
Source: Sci Rep. 2020 Sep 14;10:15042. doi: 10.1038/s41598-020-71701-8 (PMC7490348; doi:10.1038/s41598-020-71701-8)

Supplementary Materials

Title :
Humic acid has a priming effect against heat stress via transcriptional activation of HEAT-SHOCK PROTEINS in Arabidopsis

Joon-Yung Cha, Sang-Ho Kang, Imdad Ali, Sang Cheol Lee, Myung-Geun Ji, Song Yi Jeong, Gyeong-Im Shin, Min Gab Kim, Jong-Rok Jeon, Woe-Yeon Kim

Supplementary material contains two supplementary Table and six supplementary Figures.

Additional excel files are uploaded separately.
Additional file 1. Expression data of RNAseq analysis
Additional file 2. Transcript data of up-regulated genes

Supplementary Table S1. Mapping statistics for quality filtered reads generated for salt- and/or HA- treated Arabidopsis seedlings.

Treatments	Total reads and percentage	Raw	Clean	Mapped	Uniquely Mapped	Unmapped
Control-Set1	Reads	55011810	54540338	52330538	51371966	2209800
	Percenage (%)	100	0.99	0.96	0.94	0.04
Control-Set2	Reads	56532378	55876628	53613769	52799885	2262859
	Percenage (%)	100	0.99	0.96	0.95	0.04
Control-Set3	Reads	48478850	47857504	45613926	44251545	2243578
	Percenage (%)	100	0.99	0.95	0.93	0.05
HA-Set1	Reads	55928146	55130584	52546179	51857956	2584405
	Percenage (%)	100	0.99	0.95	0.94	0.05
HA-Set2	Reads	53587822	52984896	50868278	50141044	2116618
	Percenage (%)	100	0.99	0.96	0.95	0.04
HA-Set3	Reads	50687686	50089166	48099605	47477267	1989561
	Percenage (%)	100	0.99	0.96	0.95	0.04

Supplementary Table S2. Primers used in this study.

Gene name	Primer name	Primer sequence	Purpose
IAA19	IAA19-F	5′-GAGCATGGATGGTGTGCCTTAT-3′	qRT-PCR
	IAA19-R	5′-TTCGCAGTTGTCACCATCTTTC-3′	qRT-PCR
HSP101	HSP101-F	5’-GTGCGAATGTGAGAGTCCAGC-3’	qRT-PCR
	HSP101-R	5’-TCCGCACCTCTATAAGTCGAGC-3’	qRT-PCR
HSP81.1	HSP81.1-F	5’-AGTGACGATGAGGATGAAG-3’	qRT-PCR
	HSP81.1-R	5’-TTCTGCTTGTTGATGAGTTC-3’	qRT-PCR
HSP23.6-MITO	HSP23.6-F	5’-GCACACAGTTCTCAGATAA-3’	qRT-PCR
	HSP23.6-R	5’-CTACTTTCTTGTT CTCTCTT-3’	qRT-PCR
HSP17.6A	HSP17.6A-F	5’-TCAGGTCCAGATAGAGAACGAGAAC-3’	qRT-PCR
	HSP17.6A-R	5’-CCTCTCCATCCTCACAAACTTCAC-3’	qRT-PCR
AT5G12240	At5G12240-F	5′-AGCGGCTGCTGAGAAGAAAGT-3′	qRT-PCR
	At5G12240-R	5′-TCTCGAAAGCCTTGCAAAATCT-3′	qRT-PCR
TUB	TUB-F	5’-TGGCATCAACTTTCATTGGA-3’	qRT-PCR
	TUB-R	5’-ATGTTGCTCTCCGCTTCTGT-3’	qRT-PCR

Supplementary Figure S1. Cha et al.

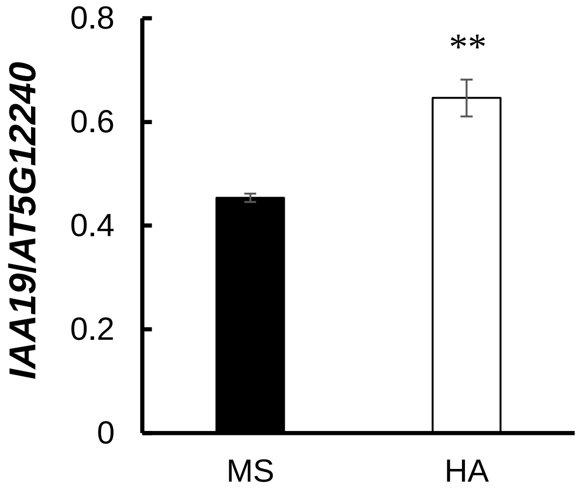

Supplementary Figure S1. Expression of *IAA19* gene transcripts responding to HA treatments in Arabidopsis seedlings.

Seedlings grown on 1/2 MS medium for 7 days were transferred onto medium with (HA) or without (MS) 860 mg L^{-1} HA for 9 h. Expression levels of *IAA19* as a marker gene for HA response were validated using qRT-PCR, and normalized to those of AT5G12240. Data represent means ± SE, n=4. Significant difference is indicated by asterisks (**p<0.01 compared to the relative expression in MS using a two-tailed Student's t-test).

Supplementary Figure S2. Cha et al.

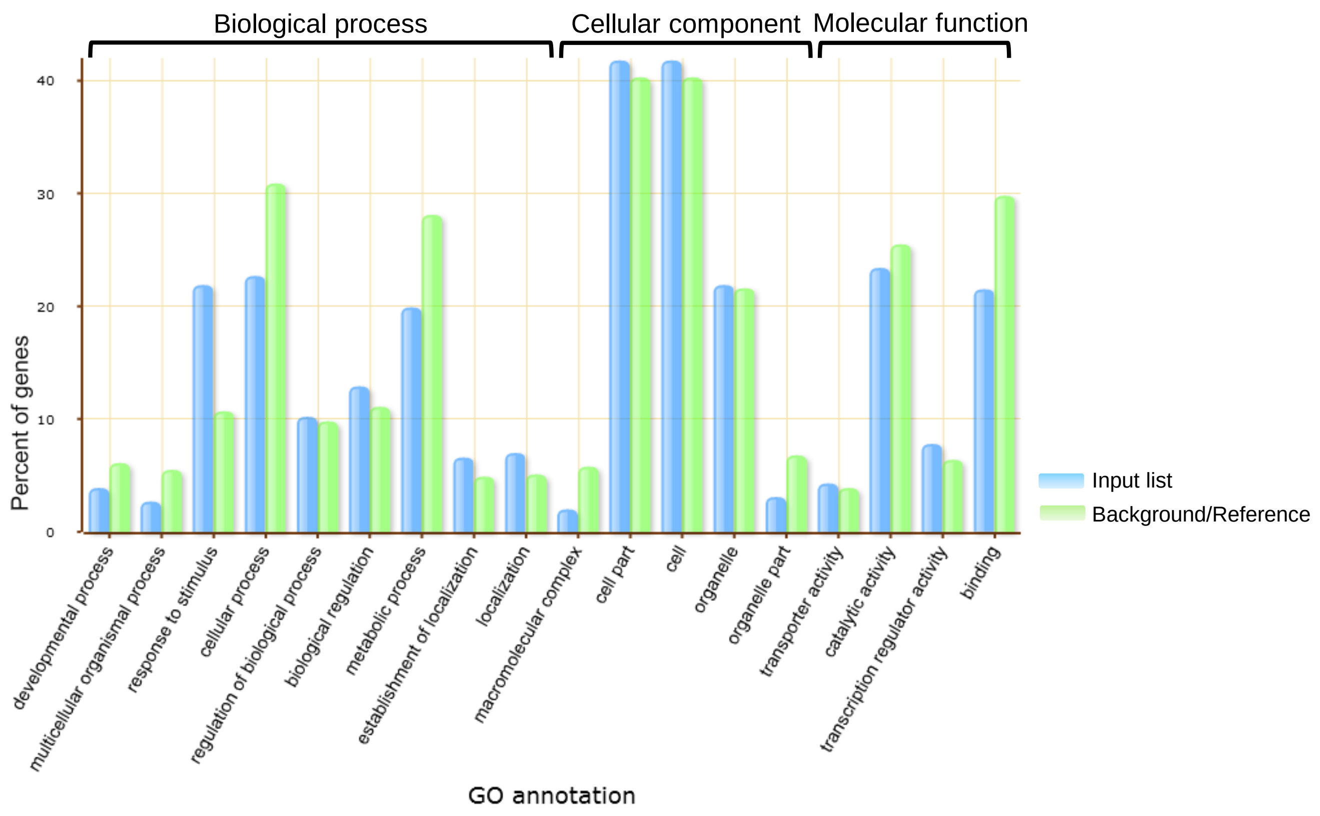

Supplementary Figure S2. GO term enrichment analysis of up-regulated genes in the main categories biological process, cellular component, and molecular function.
The percentage of genes in our input list was compared to that in the background/reference.

Supplementary Figure S3. Cha et al.

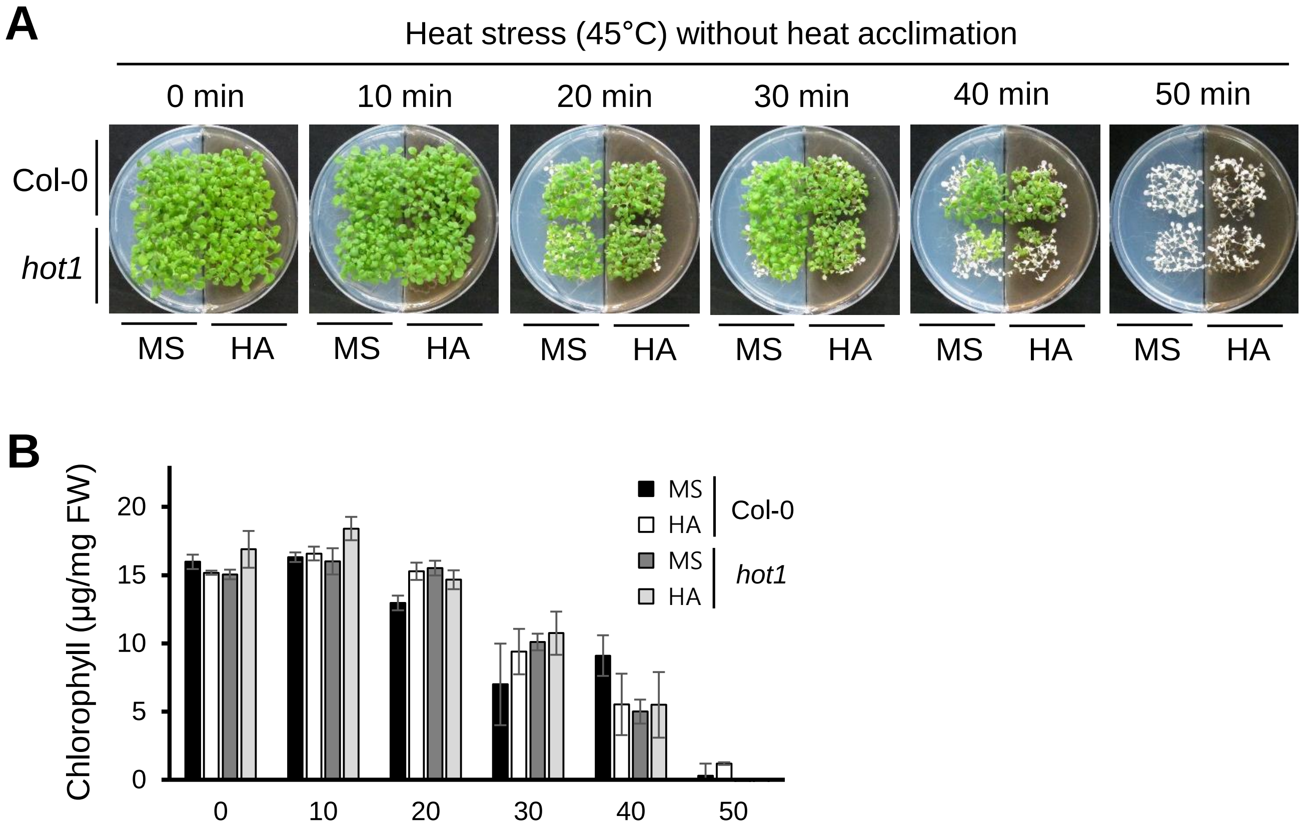

Supplementary Figure S3. Basal thermotolerance assay without heat acclimation.
Two-week old Arabidopsis wild-type (Col-0) and *hot1* mutant grown in the absence or presence of HA (860 mg L^{-1}) were exposed to heat stress at 45°C without heat acclimation. Pictures (A) were taken 5 days after heat treatment, and plants were immediately used for measuring chlorophyll contents (B). Data are means ± SE from three independent biological replications (*n*=3). Note that there are no significant differences in the chlorophyll contents of the tested lines and HA treatment.

Supplementary Figure S4. Cha et al.

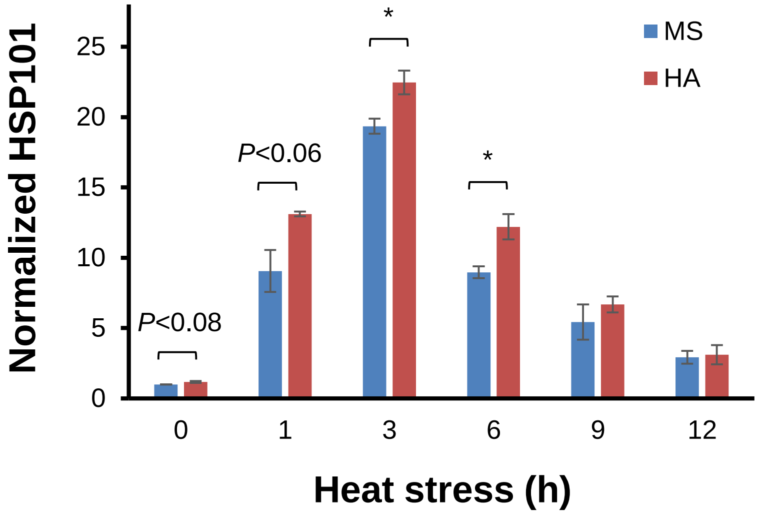

Supplementary Figure S4. Normailized HSP101 protein expression under heat stress. Two-week old Arabidopsis wild-type (Col-0) grown in the absence or presence of HA (860 mg L^{-1}) were harvested in the presence of heat stress at 45°C as shown in Fig. 5B. Immuno-blot analysis was carried out using anti HSP101 antibody. HSP101 bands from three independent biological replications were normalized by bands in CBB stained gel. Data are means ± SE (n=3). Significant differences are shown as asterisks (Student t-test, *P<0.05).

Supplementary Figure S5. Cha et al.

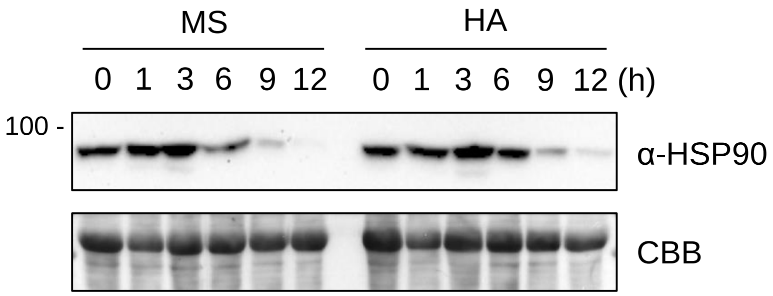

Supplementary Figure S5. HSP90 protein expression under heat stress.
Two-week old Arabidopsis wild-type (Col-0) grown in the absence or presence of HA (860 mg L^{-1}) were harvested in the presence of heat stress at 45°C as shown in Fig. 5B. Immuno-blot analysis was carried out using anti HSP90 antibody. CBB stained blot was used as a loading control. Triplicate biological replications were performed with consistent results.

Supplementary Figure S6. Cha et al.

Note: The following page (Supplementary Figure S6) shows original images of immunoblots and gels used in Figure 5 and Supplementary Figure S5 where those images were cropped.

Fig. 5A

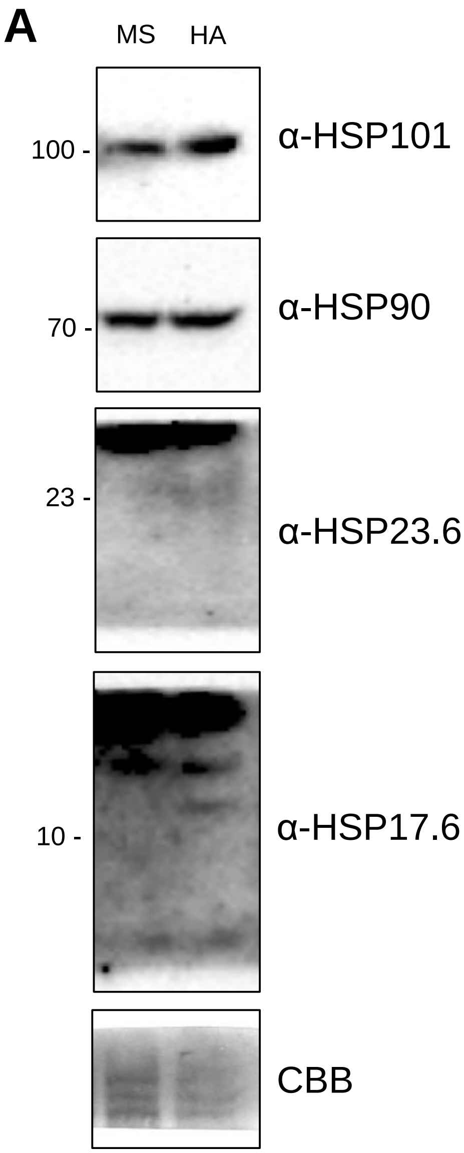

Fig. 5B

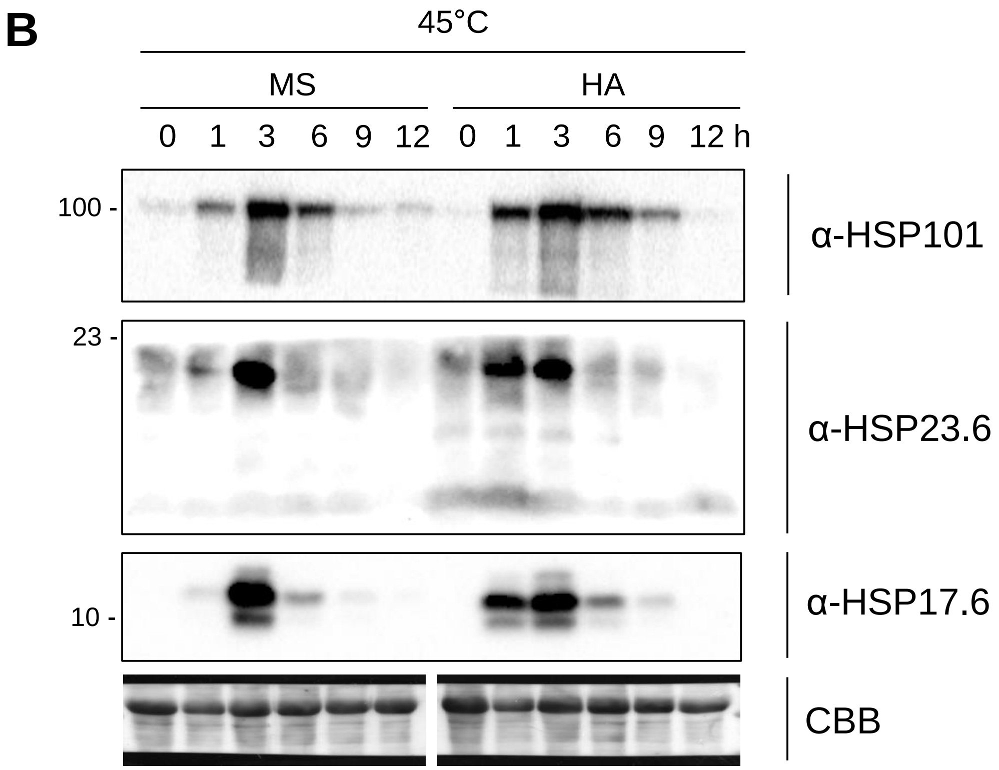
B
45°C
MS
HA
0 1 3 6 9 12 0 1 3 6 9 12 h
100 -
23 -
10 -
α-HSP101
α-HSP23.6
α-HSP17.6
CBB

Supplementary Figure S5

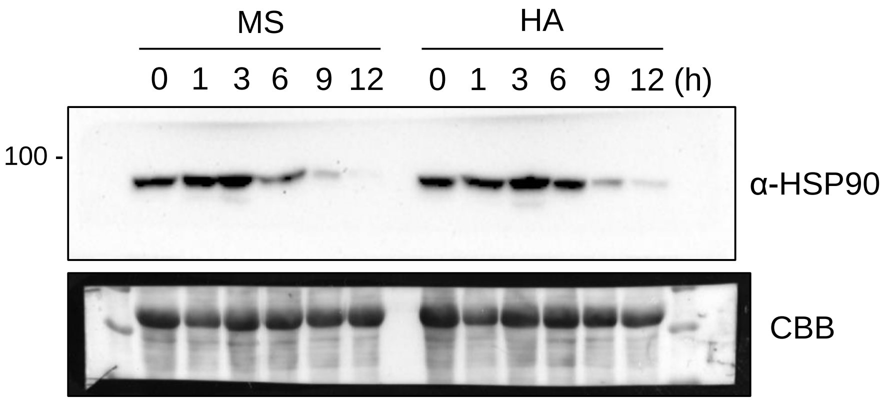

Supplement: Supplementary file 3 — Supplementary Information 3. [file 41598_2020_71701_MOESM3_ESM.pdf]
